# Darwinism and Race Progress
*"Darwinism and Race Progress." J. Berry Haycraft, M.D., D.Sc. Swan Sonnenschein. London, 1895.


**Published:** 1895-02-16

**Authors:** 


### Darwinism and Race Progress.*

"Is our civilisation a failure, or is the Caucasian played out?" Professor Haycraft, in the striking volume* before us, comes to the conclusion that there is a considerable and increasing danger of racial degeneration resulting from the conditions of modern civilisation, unless realisation of the danger bring drastic measures into play. In his argument, Dr. Haycraft sets out from Darwinian considerations. However people may differ as to the extent of the operation of selection, all are agreed that its modifying influence is a fact, not a theory. Gardeners and breeders have made use of it for generations, and have accomplished marvellous results. Individuals are born with slightly different characters; some of these characters tend to the advantage of the individuals in life, some of them to the disadvantage. Whatever be the case with differences acquired during life, it is certain that the constitutional differences, on the whole, are transmitted to the descendants of individuals. Gardeners and breeders select the individuals with variations suited to the purpose they have in view; they reject the individuals with unsuitable variations, and so, in a comparatively short space of time, alter the character and constitution of the animals or plants on which they are operating.

Dr. Haycraft applies these considerations to the human race. In past times, before preventive medicine had achieved its modern triumphs, leprosy, consumption, and most of the infectious diseases acted as friends of the human race. Little attempt being made to combat their ravages, they killed off those of weakly constitutions, chiefly in the early ages of life, and so prevented these weaker constitutions being handed on through successive generations. So also the modern success of obstetric medicine, and the greater care bestowed on children result in a large number of weakly children that in former times would have died growing up to manhood and womanhood, and producing descendants who add to the general weakness of the race. Generally speaking, the increased care now bestowed upon weakly individuals is removing a series of selective agencies which in past time kept up the standard of the constitution of the race.

Dr. Haycraft believes that the result of this change already can be traced in the death-rates. He finds that they show a diminished mortality for the earlier periods of life, but an increasing mortality for later periods; that, in fact, more of the race live to adult life, but the constitution of adults generally is lower.

Other positive factors are working to the same result. The neurotic temperament, so often tending to topple over into positive insanity, is transmitted, and persons with neurotic temperaments, in the easier conditions of modern life, quite as frequently marry and leave children as do healthy people. Similarly, the tendency to drink, not regarded by Dr. Haycraft as a specific thing, but as the index of a general nervous and mental weakness, is transmitted. Constitutional criminals and those with an inherent tendency to thriftlessness and vagabondage similarly leave as many as children do normal types. The breeder and the gardener would remove faults so great by preventing their strains from mingling with healthier strains. The human race takes no care to segregate the unfit, and their continued presence, especially now that preventive medicine has destroyed selective agencies, is a growing menace to the race.

Nor do modern forms of competition, keen enough though they be, supply the place of selective agencies. The great end of the modern struggle is for the accumulation of wealth, and, while certain sides of the brain no doubt are employed in this, it cannot be said that the successful in the struggle for wealth are mentally and constitutionally the princes of the race. There is no evidence that the modern English brain for instance is more capacious than the brain of our forefathers of the prehistoric stone age. But the actual competition is not scientifically fair; it is a handicap, and the handicaps of position, education, opportunity, wealth at starting and so forth, are scattered accidentally among the competitors. Take a practical example. Two boys of equal brain power may both aspire to the Indian Civil Service. But in actual fact one may be the son of a poor country doctor; the o·her of a rich London tradesman. The former has to struggle along, depending upon a country school, with indifferent teachers, and with a poor supply of books. The other has every educational advantage, and, say a year before the competition, he is sent to an expensive coaching institution, the staff of which has made a special study of the methods of the examiners in question, and can send the boy in without an ounce of superfluous weight in the form of knowledge that is not of immediate use for the examination. In every form of modern competition, those whom it concerns know too well the existence of this system of handicapping, and its result in securing success in the main for those favoured by start rather than by ability.

The final count in Dr. Haycraft's indictment is equally serious. He shows how on the whole the capable, the ambitious, and the successful marry later in life, and have fewer children, than the idle, the unambitious, and the less capable. In fact, it is not putting the case too strongly to say that Dr. Haycraft considers that, in the modern conditions of our civilisation, every canon is neglected by which gardeners and breeders have succeeded in maintaining and improving the choicest forms of animals and plants.

No doubt there are many flaws that could be found in the arguments, many counteracting influences that have not been taken into account; but the problem is so interesting and important that we have preferred to use the space at our disposal in stating it as clearly and incisively as we could. The time, as Dr. Haycraft says, is not yet ripe for interference of the State in problems so vast and complicated. Public opinion must be turned to the problem, aroused, and educated. For the present all private individuals must be ready to do what they can to stimulate the growing opinion against the marriage of those with congenital vices and weaknesses of constitution.

* "Darwinism and Race Progress." J. Berry Haycraft, M.D., D.Sc. Swan Sonnenschein. London, 1895.

